# A logistic regression analysis of risk factors in ME/CFS pathogenesis

**DOI:** 10.1186/s12883-019-1468-2

**Published:** 2019-11-07

**Authors:** Eliana M. Lacerda, Keith Geraghty, Caroline C. Kingdon, Luigi Palla, Luis Nacul

**Affiliations:** 1 Faculty of Infectious and Tropical Diseases, London School of Hygiene & Tropical Medicine, London, WC1E 7HT UK; 2 Centre for Primary Care, Institute of Population Health, School of Community Based Medicine, University of Manchester, Williamson Building, Brunswick Street, Manchester, M13 9PL UK

**Keywords:** Myalgic encephalomyelitis/chronic fatigue syndrome, Multiple sclerosis, Risk factors, Illness severity

## Abstract

**Background:**

Myalgic Encephalomyelitis/Chronic Fatigue Syndrome (ME/CFS) is a complex disease, whose exact cause remains unclear. A wide range of risk factors has been proposed that helps understanding potential disease pathogenesis. However, there is little consistency for many risk factor associations, thus we undertook an exploratory study of risk factors using data from the UK ME/CFS Biobank participants. We report on risk factor associations in ME/CFS compared with multiple sclerosis participants and healthy controls.

**Methods:**

This was a cross-sectional study of 269 people with ME/CFS, including 214 with mild/moderate and 55 with severe symptoms, 74 people with multiple sclerosis (MS), and 134 healthy controls, who were recruited from primary and secondary health services. Data were collected from participants using a standardised written questionnaire. Data analyses consisted of univariate and multivariable regression analysis (by levels of proximity to disease onset).

**Results:**

A history of frequent colds (OR = 8.26, *P* <= 0.001) and infections (OR = 25.5, *P* = 0.015) before onset were the strongest factors associated with a higher risk of ME/CFS compared to healthy controls. Being single (OR = 4.41, *P* <= 0.001), having lower income (OR = 3.71, *P* <= 0.001), and a family history of anxiety is associated with a higher risk of ME/CFS compared to healthy controls only (OR = 3.77, P < 0.001). History of frequent colds (OR = 6.31, *P* < 0.001) and infections before disease onset (OR = 5.12, *P* = 0.005), being single (OR = 3.66, *P* = 0.003) and having lower income (OR = 3.48, *P* = 0.001), are associated with a higher risk of ME/CFS than MS. Severe ME/CFS cases were associated with lower age of ME/CFS onset (OR = 0.63, *P* = 0.022) and a family history of neurological illness (OR = 6.1, *P* = 0.001).

**Conclusions:**

Notable differences in risk profiles were found between ME/CFS and healthy controls, ME/CFS and MS, and mild-moderate and severe ME/CFS. However, we found some commensurate overlap in risk associations between all cohorts. The most notable difference between ME/CFS and MS in our study is a history of recent infection prior to disease onset. Even recognising that our results are limited by the choice of factors we selected to investigate, our findings are consistent with the increasing body of evidence that has been published about the potential role of infections in the pathogenesis of ME/CFS, including common colds/flu.

## Background

Myalgic Encephalomyelitis (ME) was originally described as a post-infectious disease causing malaise, muscle weakness, and nervous system complaints, primarily pain, cognitive dysfunction, and sleep disturbance [1]. Chronic fatigue syndrome (CFS) is an alternative label introduced in the late 1980s to describe a pattern of symptoms, specifically unexplained fatigue [2]. The two names are often used synonymously. ME/CFS prevalence rates vary widely across studies, but a rate between 0.2 and 0.5% is commonly reported for adults [3]**.** A number of different diagnostic criteria are used to identify potential cases. In the UK, the National Institute for Health and Care Excellence (NICE) has recommended a diagnosis after 6 months of persistent unexplained fatigue, not relieved by rest, which results in a substantial loss of normal physical or social function [4]. The US Centre for Disease Control (CDC) criteria from 1994 require a wider set of characteristic symptoms [5], whilst other criteria require the presence of post-exertional malaise [6]. The aetiology and pathogenesis of ME/CFS remains contested but many patients recount their symptoms starting after an infection and an increasing number of studies find neuro-immunological and cellular abnormalities that support an association between infection and pro-inflammatory immune alterations in ME/CFS [7, 8].

A range of disparate risk factors has been proposed as disease-specific. A number of studies suggest a higher prevalence of ME/CFS among family members, particularly twins [9], suggesting a genetic heritability risk factor. Underhill and O’Gorman found that 20.5% of members of a US CFS sample, reported a family member with CFS (18% being blood relatives), suggesting a strong genetic predisposition [10]. A genetic study has found a number of DNA single-nucleotide polymorphisms (SNPs) from over 906,600 known SNPs analysed from ME/CFS subjects, and identified 442 potential loci that might be associated with ME/CFS [11]. Despite the small sample size, this study exemplifies the vast complexity of genes as a risk factor in ME/CFS. The relative-risk attached to such factors is difficult to ascertain from a review of the literature. A systematic scoping review by Hempel et al. analysed risk factors for ME/CFS using multiple predictors [12], but from 10,768 relevant publications, only 11 met inclusion criteria. Hempel et al. concluded that there was poor replication of risk factors across multiple studies, so that few demographic, medical, psychological, social and environmental factors can be considered suitable predictive indicators in clinical practice. A major problem in the studies reviewed is the variability of diagnostic criteria used (including in self-reported ME/CFS) and a lack of consistent methodology. The most credible risk factors for ME/CFS onset are sex, with a higher female to male ratio [13] and a history of infection [14], the latter highlighting the importance of environmental factors in the aetiology of ME/CFS, which may act independently or interact with genetic risk factors. Large population datasets have been used to explore pre-morbid health factors and ME/CFS. There is some evidence of a link between affective disorders (anxiety and depression) and ME/CFS while work on pre-morbid activity levels has not been able to establish a firm link with prior levels of physical activity. A link between CFS and childhood abuse has been suggested, although results from case-control studies have been contradictory [15, 16].

The need to further explore and assess risk factors for ME/CFS prompted this study, which investigates potential risk factors by comparing data from a cohort of the UK ME/CFS Biobank (UKMEB) participants. This cohort includes people with ME/CFS, people with multiple sclerosis (MS), and healthy controls.

## Methods

The UKMEB team has collected patient data and biological samples from informed consenting participants since 2012. Recruitment procedures for the UKMEB have been exemplified elsewhere, in a publication that also lists the data collection instruments used [17]. Recruitment for the UKMEB cohort included the invitation of potential participants by collaborating NHS Services (primary and secondary care), who used their databases to identify people diagnosed with ME/CFS, people diagnosed with MS, and potential healthy controls, aged between 18 and 60 years. The NHS Services sent out invitation packs provided by the research team containing an invitation letter from the health service with information about the study (with specific information sheets for cases and controls), a consent form, a questionnaire to assess symptoms, and a refusal form. People with ME/CFS who are bed- or home-bound are often unable to attend the NHS services, and were invited by support groups. Health services and higher education institutions such as the LSHTM, also handed invitation packs for potential healthy controls.

Once signed consent forms and questionnaires had been assessed by the research team, those who had a likely diagnosis of ME/CFS according to the research criteria (CDC-1994 [5] or Canadian Consensus Criteria [6]) and who were able to travel were invited to a recruiting centre by the research team, while those with severe disease and mobility restrictions were visited at home by a clinical researcher. Participants were excluded if they had: i) used drugs known to alter immune function (e.g. azathioprine, cyclosporine, methotrexate, steroids), anti-viral medications and vaccinations in the 3 months prior to recruitment; or ii) a history of acute or chronic infectious diseases such as hepatitis B and C, tuberculosis, HIV (but not herpes virus or other retrovirus infection); iii) a history of other severe illness (such as cancer, coronary heart disease, or uncontrolled diabetes), and/or and severe mood disorders, iv) a history of illicit drug use; and/or v) a BMI ≥ 40. Pregnant women and those within 12 months post-partum and/or currently lactating were also excluded. Those people who had offered to take part but were ineligible were thanked by the research manager and a full explanation of the reason was given.

At the clinical appointment, all participants were examined by a health professional; the diagnosis of ME/CFS for research purposes was reached only after this assessment and following the results of the clinical blood tests taken, which were aimed to exclude other conditions that could explain chronic fatigue. All participants with MS had a prior diagnosis from a UK NHS neurology consultant according to NHS guidelines [18].

We invited 2430 individuals identified by our collaborating NHS services (942 with ME/CFS, 278 with MS and 1210 healthy), in addition to 112 people with a confirmed medical diagnosis of ME/CFS invited by ME/CFS support groups, of whom 84 invited healthy individuals to act as controls. Of the total potential participants invited, 138 declined to participate (45 had a possible diagnosis of ME/CFS, 26 MS, 48 healthy controls; 19 received refusal forms were incomplete) and 1828 were non-respondents. The distribution by sex and age group of those who declined to participate in all groups, was similar to the groups of those recruited, and the proportion of stated refusals was similar across the recruiting health services varying between 4 and 10% (median 6.3%, IQR 5.3 to 8.9%). From the consenting potential participants, 660 were assessed for eligibility as previously described, of which 532 were recruited. After additional exclusions, per study protocol, the final cohort considered in this paper includes ME/CFS participants with mild/moderate (*n*-214), and with severe symptoms (*n*-55), participants with MS (*n*-74), and healthy controls (*n*-134).

### Data analysis

UKMEB participant questionnaire responses [17] were grouped under the following headings: socio-economic, demographic, family health history, lifestyle, co-morbidities, and other potential risk factors associated with ME/CFS (See Additional file 1). All these were self-reported, as we did not have access to their medical records to further explore the presence of these risk factors. Due to the cross sectional design of the study, with control groups (where controls are either healthy or MS subjects), logistic regression was used for prediction (binary outcome, logit link, structural linear model, with model parameters estimated by maximum likelihood). Because of the limited sample size / number of cases and the presence of a large number of predictors within a logistic regression prediction model, the framework used for analysis considered separate variable domains for prediction of the outcome (ME/CFS cases) versus one of the two comparison groups (i.e. people with MS, and healthy controls), in order to select the predictors. The analysis framework was inspired by a conceptual approach to risk factor modelling according to which risk factors can be separated into distinct hierarchical levels relative to the outcome [18].

After running univariate logistic regression analyses for each putative risk factor in all domains (Table 1), we included in the multivariable logistic regression models those factors that showed a statistically significant difference with the comparison group (*P* ≤ 0.10). The following variables from the recent exposures domain ‘immunisations’ and ‘BCG vaccination’ were later aggregated as one variable named ‘immunisation(s) before onset’; likewise, ‘meningitis’ and ‘other serious infection’, were aggregated into a new variable named ‘infection(s) before onset’. Subsequently, we ran the multivariable models, starting with the more distal domain (demographic) and working towards the proximate domain (recent exposures) to the outcome. The model selection strategy was to add all the variables of the subsequent domain and, in a step-wise manner, remove those whose likelihood ratio test (comparing reduced and full model) had *P*-value> 0.05. The overall model fit (Pearson chi squared test, pseudo R squared) and predictive ability (sensitivity/specificity, correct classification rate) were also assessed for all models. The analyses were conducted with complete cases. The analyses were performed with Stata 15.1 [20].

**Table 1:** List of variables used in the univariate logistic regression analyses with all comparison groups, organised by domains

Domains	List of variables
Demographic	Age at survey (or age at disease onset for the diseased groups), sex, ethnicity.
Socio-economic	Marital status, education, individual income, index of multiple deprivation^a^.
Family health history	High blood pressure, diabetes, heart problems, asthma, allergies, depression, anxiety, mental health problems, learning disabilities, physical disabilities, cancer, ME/CFS, MS, other neurological problems, tuberculosis, any other health problems.
Lifestyle	Smoking status, alcohol consumption/week, physical activity
Previous morbidity (prior to disease onset)	High blood pressure, heart problems, asthma, allergies, depression, anxiety, high levels of stress, history of frequent coughs/colds, intolerance to alcoholic drinks, intolerance to sugar, other health problems.
Recent exposures (6 months prior to disease onset)	Lived on a farm, tick bites, vaccines/immunisations, travelling overseas, flooding, radiation, carbon monoxide (CO), pesticides, any other chemicals, had meningitis, had any other serious infection, had any head injury, had a major operation, contraceptive pill (women only), lived in a house with gas or oil appliances (for heating or cooking), lived in a house with a gas fire, BCG vaccination.

*ME/CFS* Myalgic Encephalomyelitis/Chronic Fatigue Syndrome, *MS* Multiple Sclerosis.

^a^Index of multiple deprivation refers to area of residence [19]. All other variables are as reported by research participants

## Results

We found a 3:1 female to male ratio in our ME/CFS and MS participant groups; however, the female/male ratio in the healthy control group was 1.5:1 (*P* = 0.011). The age group distribution was similar among the healthy and ME/CFS groups (*P* = 0.943); the MS group had a higher proportion of individuals over 30 years of age (*P* = 0.002). The most common ethnicity reported by participants in all groups was white British (> 90%), with the groups of ME/CFS with severe symptoms and of healthy controls reporting a slightly more diverse ethnic background which still amounted to a small proportion of participants (< 10%).

In Table 2 we present a description of participants from the UK ME/CFS Biobank, by category of recruitment and each distinct domain, containing distinct sets of variables.

**Table 2:** Description of participants from the UK ME/CFS Biobank, by category of recruitment and variables under the following domains: demographic, socio-economic, family health history, lifestyle, previous morbidity (prior disease onset for the diseased groups), and recent exposures

Domains	Variables		Category of Recruitment						Total	
			Healthy control		MS control		ME/CFSmm			
			(n)	(%)	(n)	(%)	(n)	(%)	(n)	(%)
Demographic	Sex	Male	51	38.1	16	21.6	63	23.4	130	27.3
		Female	83	61.9	58	78.4	206	76.6	347	72.7
	Age group	>=18 & <30	22	16.4	1	1.4	43	16.0	66	13.8
		>=30 & <40	33	24.6	12	16.2	60	22.3	105	22.0
		>=40 & <50	35	26.1	18	24.3	75	27.9	128	26.8
		>=50 & <=60	44	32.8	43	58.1	91	33.8	178	37.3
	Incidence age group	<18	n/a	n/a	3	4.2	42	16.7	45	14.0
		>=18 & <30	n/a	n/a	19	26.8	88	35.1	107	33.2
		>=30 & <40	n/a	n/a	22	31.0	61	24.3	83	25.8
		>=40 & <50	n/a	n/a	22	31.0	49	19.5	71	22.0
		>=50 & <=60	n/a	n/a	5	7.0	11	4.4	16	5.0
	Ethnicity	White	119	91.5	70	94.6	247	96.5	436	94.8
		Other	11	8.5	4	5.4	9	3.5	24	5.2
Socio-economic	Current marital status	Married/with partner	98	76.6	52	72.2	141	54.9	291	63.7
		Single	30	23.4	20	27.8	116	45.1	166	36.3
	Marital status (prior disease onset)	Married/with partner	n/a	n/a	56	76.0	115	44.8	171	51.8
		Divorced/separated	n/a	n/a	5	6.7	26	10.1	31	9.4
		Single	n/a	n/a	12	17.3	116	45.1	128	38.8
	Education	Up to high school	37	28.9	28	38.9	93	36.5	158	34.7
		Started HE	13	10.2	11	15.3	40	15.7	64	14.1
		Completed HE/PG	78	60.9	33	45.8	122	47.8	233	51.2
	Individual income (current)	Higher than £19,999	63	51.6	28	43.8	40	16.7	131	30.8
		Up to £19,999	59	48.4	36	56.2	199	83.3	294	69.2
	Individual income (prior disease onset)	Higher than £19,999	n/a	n/a	36	56.2	67	28.8	103	34.7
		Up to £19,999	n/a	n/a	28	43.8	166	71.2	194	65.3
			mean	SD	mean	SD	mean	SD		
	Index of multiple deprivation^a^	Deciles	5.9	2.6	5.8	2.6	5.8	2.6		
			(n)	(%)	(n)	(%)	(n)	(%)	(n)	(%)
Family health history	High blood pressure	No	69	54.3	39	56.5	116	47.4	224	50.8
		Yes	58	45.7	30	43.5	129	52.7	217	49.2
	Diabetes	No	102	81.0	49	70.0	178	73.6	329	75.1
		Yes	24	19.1	21	30.0	64	26.5	109	24.9
	Heart problems	No	85	68.0	44	62.0	147	59.8	276	62.4
		Yes	40	32.0	27	38.0	99	40.2	166	37.6
	Asthma	No	93	74.4	48	68.6	140	58.3	281	64.6
		Yes	32	25.6	22	31.4	100	41.7	154	35.4
	Allergies	No	72	57.6	49	72.1	98	40.8	219	50.6
		Yes	53	42.4	19	27.9	142	59.2	214	49.4
	Depression	No	80	64.0	48	66.7	132	54.3	260	59.1
		Yes	45	36.0	24	33.3	111	45.7	180	40.9
	Anxiety	No	101	80.8	49	70.0	131	54.4	281	64.5
		Yes	24	19.2	21	30.0	110	45.6	155	35.6
	Mental health problems	No	106	84.8	59	83.1	182	75.8	347	79.6
		Yes	19	15.2	12	16.9	58	24.2	89	20.4
	Learning disabilities	No	93	97.9	66	93.0	127	88.2	286	92.3
		Yes	2	2.1	5	7.0	17	11.8	24	7.7
	Physical disabilities	No	88	92.6	62	87.3	120	83.9	270	87.4
		Yes	7	7.4	9	12.7	23	16.1	39	12.6
	Cancer	No	77	61.1	30	41.7	144	60.0	251	57.3
		Yes	49	38.9	42	58.3	96	40.0	187	42.7
	ME/CFS	No	117	94.4	67	97.1	214	89.9	398	92.3
		Yes	7	5.7	2	2.9	24	10.1	33	7.7
	MS	No	94	99.0	60	85.7	137	97.9	291	95.4
		Yes	1	1.1	10	14.3	3	2.1	14	4.6
	Other neurological problems	No	87	91.6	64	90.1	117	83.0	268	87.3
		Yes	8	8.4	7	9.9	24	17.0	39	12.7
	Tuberculosis	No	93	97.9	61	88.4	136	95.8	290	94.8
		Yes	2	2.1	8	11.6	6	4.2	16	5.2
	Any other health problems	No	91	73.4	48	68.6	107	45.7	246	57.5
		Yes	33	26.6	22	31.4	127	54.3	182	42.5
Lifestyle			median	IQR	median	IQR	median	IQR		
	Alcohol consumption	Currently (units/week)	4	1 - 10	2	0 - 9	0	0 - 2	n/a	n/a
	Alcohol consumption	Prior disease onset (units/week)	n/a	n/a	3	0 - 10	2	0 - 7	n/a	n/a
			(n)	(%)	(n)	(%)	(n)	(%)	(n)	(%)
	Smoking status	Never smoke	85	65.4	30	40.5	159	62.1	274	59.6
		Ex-smoker	39	30.0	26	35.1	66	25.8	131	28.5
		Current smoker	6	4.6	18	24.3	31	12.1	55	12.0
	Physical activity level	Rather inactive/not active	19	14.6	7	9.5	13	5.1	39	8.5
		Neither active nor inactive	23	17.7	11	14.9	23	9.0	57	12.4
		Very active/rather active	88	67.7	56	75.7	219	85.9	363	79.1
Previous morbidity	High blood pressure	No	118	90.8	68	95.8	240	95.2	426	94.0
		Yes	12	9.2	3	4.2	12	4.8	27	6.0
	Heart problems	No	125	96.9	70	98.6	242	96.0	437	96.7
		Yes	4	3.1	1	1.4	10	4.0	15	3.3
	Asthma	No	115	90.6	66	91.7	187	74.2	368	81.6
		Yes	12	9.5	6	8.3	65	25.8	83	18.4
	Allergies	No	67	51.9	49	67.1	143	57.7	259	57.6
		Yes	62	48.1	24	32.9	105	42.3	191	42.4
	Depression	No	113	86.9	57	78.1	157	62.1	327	71.7
		Yes	17	13.1	16	21.9	96	37.9	129	28.3
	Anxiety	No	100	76.9	57	78.1	153	60.2	310	67.8
		Yes	30	23.1	16	21.9	101	39.8	147	32.2
	High levels of stress	No	75	57.7	36	50.7	106	42.4	217	48.1
		Yes	55	42.3	35	49.3	144	57.6	234	51.9
	Frequent coughs/colds	No	122	93.9	62	88.6	155	61.8	339	75.2
		Yes	8	6.2	8	11.4	96	38.3	112	24.8
	Intolerance to alcoholic drinks	No	121	94.5	71	100.0	213	88.4	405	92.1
		Yes	7	5.5	0	0.0	28	11.6	35	8.0
	Intolerance to sugar	No	129	99.2	72	100.0	235	94.8	436	96.9
		Yes	1	0.8	0	0.0	13	5.2	14	3.1
	Other health problems	No	111	89.5	60	89.6	166	81.8	337	85.5
		Yes	13	10.5	7	10.5	37	18.2	57	14.5
Recent exposures	Lived on a farm	No	126	96.9	68	95.8	238	94.1	432	95.2
		Yes	4	3.1	3	4.2	11	4.4	18	4.0
		Don't know	0	0.0	0	0.0	4	1.6	4	0.9
	Tick bites	No	71	98.6	25	96.2	78	86.7	174	92.6
		Yes	0	0.0	0	0.0	1	1.1	1	0.5
		Don't know	1	1.4	1	3.9	11	12.2	13	6.9
	Vaccines/immunisations	No	67	93.1	19	73.1	80	88.9	166	88.3
		Yes	5	6.9	7	26.9	8	8.9	20	10.6
		Don't know	0	0.0	0	0.0	2	2.2	2	1.1
	Travelled overseas	No	69	53.1	39	53.4	150	59.3	258	56.6
		Yes	61	46.9	31	42.5	87	34.4	179	39.3
		Don't know	0	0.0	3	4.1	16	6.3	19	4.2
	Flooding	No	128	98.5	73	98.7	250	97.7	451	98.0
		Yes	2	1.5	1	1.4	3	1.2	6	1.3
		Don't know	0	0.0	0	0.0	3	1.2	3	0.7
	Radiation	No	71	98.6	25	96.2	82	91.1	178	94.7
		Yes	1	1.4	1	3.9	4	4.4	6	3.2
			0	0.0	0	0.0	4	4.4	4	2.1
	Carbon monoxide (CO)	No	128	97.7	68	89.5	210	81.4	406	87.3
		Yes	1	0.8	0	0.0	3	1.2	4	0.9
		Don't know	2	1.5	8	10.5	45	17.4	55	11.8
	Pesticides	No	119	92.3	57	77.0	174	68.2	350	76.4
		Yes	2	1.6	2	2.7	18	7.1	22	4.8
		Don't know	8	6.2	15	20.3	63	24.7	86	18.8
	Any other chemicals	No	118	91.5	54	73.0	158	62.2	330	72.2
		Yes	6	4.7	3	4.1	25	9.8	34	7.4
		Don't know	5	3.9	17	23.0	71	28.0	93	20.4
	Had meningitis	No	130	100.0	73	98.7	247	96.5	450	97.8
		Yes	0	0.0	1	1.4	2	0.8	3	0.7
		Don't know	0	0.0	0	0.0	7	2.7	7	1.5
	Had any other serious infection	No	128	98.5	65	89.0	167	67.9	360	80.2
		Yes	2	1.5	6	8.2	62	25.2	70	15.6
		Don't know	0	0.0	2	2.7	17	6.9	19	4.2
	Had any head injury	No	129	100.0	65	89.0	229	89.8	423	92.6
		Yes	0	0.0	6	8.2	16	6.3	22	4.8
		Don't know	0	0.0	2	2.7	10	3.9	12	2.6
	Had a major operation	No	128	99.2	68	91.9	235	92.2	431	94.1
		Yes	1	0.8	6	8.1	15	5.9	22	4.8
		Don't know	0	0.0	0	0.0	5	2.0	5	1.1
	Contraceptive pill (women only)	No	67	52.8	33	45.8	139	55.4	239	53.1
		Yes	16	12.6	23	31.9	63	25.1	102	22.7
		Don't know	3	2.4	0	0.0	4	1.6	7	1.6
	Lived in a house with gas or oil appliances	No	28	21.5	24	32.9	50	19.8	102	22.4
		Yes	102	78.5	49	67.1	201	79.8	352	77.4
		Don't know	0	0.0	0	0.0	1	0.4	1	0.2
	Lived in a house with a gas fire	No	100	77.5	46	63.9	153	61.0	299	66.2
		Yes	29	22.5	26	36.1	97	38.7	152	33.6
		Don't know	0	0.0	0	0.0	1	0.4	1	0.2
	BCG vaccination	No	17	17.4	24	32.9	26	17.1	67	20.7
		Yes	63	64.3	41	56.2	97	63.8	201	62.2
		Don't know	18	18.4	8	11.0	29	19.1	55	17.0

MS – multiple sclerosis; ME/CFS - Myalgic Encephalomyelitis/Chronic Fatigue Syndrome; HE – Higher education; HE/ PG - Higher education/post-graduation;

^a^Index of multiple deprivation (IDM) is the measure of relative deprivation for small areas in England (official measure). It is composed by the following indices: Income Deprivation (22.5%), Employment Deprivation (22.5%), Education, Skills and Training Deprivation (13.5%), Health Deprivation and Disability (13.5%), Crime (9.3%), Barriers to Housing and Services (9.3%), and Living Environment Deprivation (9.3%). IDM decile 1 refers to the most deprived area and decile 10 to the least deprived area (https://assets.publishing.service.gov.uk/government/uploads/system/uploads/attachment_data/file/464430/English_Index_of_Multiple_Deprivation_2015_-_Guidance.pdf)

Table 3 shows the variables associated with the outcome in each domain level (*P* < 0.10), with comparisons between ME/CFS cases and healthy controls, and ME/CFS and MS cases.

**Table 3:** List of variables included in the multivariable analyses, by comparison groups and levels of hierarchy

Domain	Significant variables resulting from the bivariate analyses, included in the multivariable models (*p* ≤ 0.10)
Multivariable analysis 1 – Comparison between people with ME/CFS and healthy controls	
Level 1 - Demographic	Age at survey, sex, ethnicity
Level 2 - Socio-Economic	Marital status, education, income
Level 3 - Family Health History	Asthma, allergies, depression, anxiety, learning disabilities, mental health issues, physical disabilities, other health problems, other neurologic problems
Level 4 - Lifestyle	Smoking status, alcohol consumption/week, physical activity previous 6 months
Level 5 - Previous Morbidity (prior to disease onset)	Asthma, depression, anxiety, stress, colds, alcohol intolerance, sugar intolerance, other health problems
Level 6 - Recent Exposures (6 months prior to disease onset)	Travelling, pesticides, chemicals, infection(s), pill contraception, surgery(ies), and gas fire appliances in the home
Multivariable Analysis2 – Comparison between people with ME/CFS and people with MS	
Level 1 - Demographic	Age at disease onset, sex, ethnicity
Level 2 - Socio-Economic	Marital status, education, income, and occupation at 6 months previous onset
Level 3 - Family Health History	Allergies, anxiety
Level 4 - Lifestyle	Alcohol consumption/week, physical activity previous 6 months
Level 5 - Previous Morbidity (prior to disease onset)	Asthma, allergies, depression, anxiety, stress, colds, alcohol intolerance, sugar intolerance, other health problems
Level 6 - Recent Exposures (6 months prior to disease onset)	Infection, BCG vaccine

*ME/CFS* Myalgic Encephalomyelitis/Chronic Fatigue Syndrome, *MS* Multiple Sclerosis.

From the multivariable logistic regression analysis for each of the comparisons, we found that compared to healthy controls, participants with ME/CFS were less likely to be in a relationship (be single), more likely to have a lower income, to report a family history (but not a personal history) of anxiety, and to report frequent colds and coughs, and infections in the 6 months prior to disease onset (Table 4). The model fit statistics resulted in Pearson chi-square *P* = 0.80 and pseudo R squared = 0.33, 78% of individuals correctly classified, sensitivity of 83% and specificity of 71%. Similarly, by comparing ME/CFS and MS participants, we found increased risks of ME/CFS related to not being in a relationship, have a lower income, have a history of predisposition to colds and coughs, and to having an infectious disease in the 6 months before disease onset (Table 5). This model had worse model fit statistics with Pearson chi-square *P* = 0.004 and pseudo R squared = 0.26, 82% of individuals correctly classified, sensitivity of 95% and specificity of 34%.

**Table 4:** Final multivariable model, comparing participants with ME/CFS and healthy controls (*n* = 324)

Variables	Odds Ratio	Standard Error	95% Confidence Interval	*P* value
Age at survey (in years)	1.05	0.01	1.02–1.08	0.003
Lower income^a^	3.71	1.18	1.10–6.92	< =0.001
Marital status – separated or divorced^b^	1.84	1.02	0.62–5.47	0.275
Marital status – single^b^	4.41	1.77	2.00–9.71	<= 0.001
Family history of anxiety	3.77	1.23	1.98–7.16	<= 0.001
History of frequent colds/flu	8.26	3.46	3.64–18-77	<= 0.001
Infection(s) 6 months before disease onset	25.5	20.3	5.33–121.76	0.015

*ME/CFS* Myalgic Encephalomyelitis/Chronic Fatigue Syndrome.

^a^Up to Up to £19,999/year; ^b^the refence category is married/with partner, i.e. in a stable relationship

**Table 5:** Final multivariable model, comparing participants with ME/CFS and participants with MS (*n* = 273)

Variables	Odds Ratio	Standard Error	95% Confidence Interval	*P* value
Age at disease onset (in years)	0.95	0.02	0.92–0.99	0.016
Lower income^a^	3.48	1.27	1.70–7.13	0.001
Marital status – separated or divorced^b^	2.26	1.61	0.56–9.12	0.250
Marital status – single^b^	3.66	1.62	1.54–8.72	0.003
History of frequent colds/flu	6.31	3.16	2.36–16.86	< 0.001
Infection(s) 6 months before disease onset	5.12	2.97	1.66–15.98	0.005

*ME/CFS* Myalgic Encephalomyelitis/Chronic Fatigue Syndrome.

^a^Up to Up to £19,999/year; ^b^the refence category is married/with partner, i.e. in a stable relationship

Those with severe ME/CFS were more likely to be younger; 15 of these participants reported a family history of neurological problems, of which the most commonly reported were stroke (4) and Parkinson’s disease (3/15); 9/15 reported that their father was affected and 4/15, their mother. Of the 9 people with mild-moderate ME/CFS who reported neurological family problems, 4 of those reported had a family history of dementia, 5 reported that it was their mother who was affected.

## Discussion

Our recruited UKMEB cohort reflects the predominance of ME/CFS and MS in females that has been reported in the literature. There is consistent evidence for a higher rate of ME/CFS among girls and women [21], with rates among girls increasing above those of boys post-puberty [22]. A Spanish study of disease epidemiology among 1309 CFS patients meeting the Fukuda criteria found a 90% female dominance [23], however ratios between 2:1 to 4:1 are often reported [3]. This female dominance is not uncommon in autoimmune diseases; MS affects more women than men in a similar ratio [24]. In terms of epidemiological and neuro-immune characteristics, associations have been drawn between ME/CFS and MS, fibromyalgia, and rheumatoid arthritis [25]. However, we also must consider that the majority of our cohort was recruited from primary/secondary care services, which have been reported to have higher attendance of females, particularly between 16 and 60 years of age, when the gender gap was observed in the UK [26], The gender differences for health care seeking varies greatly across populations [26, 27], and we must take these variations into account when interpreting study results that recruit from health services.

### ME/CFS v healthy controls

Most participants with ME/CFS anecdotally report their illness started after an infection [28] and our study affirms the importance of infection as a strong risk factor for ME/CFS onset. Our findings indicate that a history of frequent colds and infection in the 6 months preceding disease onset is associated with a higher risk of ME/CFS, compared with healthy controls and participants with MS. Research has shown that ME/CFS is linked with exposure to Epstein–Barr virus, Coxsackie B, Human Herpes virus 6 and 7, and *Coxiella burnetii* [14, 29, 30], with stronger associations with infections in those with more severe acute response to infections [31]. Chia and Chia proposed a link between ME/CFS and enterovirus infection after the biopsies from 135/165 CFS patients (82%) stained positive for VP1 within parietal cells, versus just 7/34 (20%) of healthy controls [32]. There is scant research linking ME/CFS to the common cold or flu-like infections, though upper respiratory infections are often reported as preceding the development of disabling fatigue in clinical practice. Our findings suggest a risk association, based on self-report. Clark et al. found a history of colds in childhood (at age 7 or 11) increased the risk of ME/CFS later in life (ORs ranged from 1.6 to 1.9) [33]. The predictive role of pre-morbid stress and infection is frequently reported in ME/CFS [34]. The exact cumulative impact of these two factors is uncertain, although it is well established that chronic stress has a considerable depressive effect on immune status, perhaps rendering an individual more susceptible to chronic infection. It is known that herpes viruses (HSV1 and HSV2, HHV6) are associated with a range of acute and chronic illnesses including, encephalitis/meningitis, shingles, chicken pox (Varicella Zoster), mononucleosis (Epstein Barr Virus), Kaposi’s sarcoma (HHV8); and hearing loss, mental retardation with cytomegalovirus (HMCV) [30, 35]. Viral infections may disrupt mitochondrial function, resulting in fatigue; a cardinal symptom of ME/CFS, and initiate an array of inflammatory responses in ME/CFS [7, 8] that help explain the pain and other symptoms experienced by people with ME/CFS. Two studies reveal clustering of cases in UK schools [36, 37] giving credence to the role of infectious aetiology in ME/CFS in adolescents/children. Historically there have been reports of ME-type cluster outbreaks in adults in defined geographical areas, such as Los Angeles (*poliomyelitis* - 1934), Iceland (1948 - *Icelandic disease*), Royal Free Disease, (London, UK 1955 - *benign myalgic encephalomyelitis*), Florida (*epidemic neuromyasthenia* - 1957), New Zealand (*Tapanuni flu* – 1983), Nevada (Lake Tahoe 1984 - *chronic fatigue syndrome*). A more recent outbreak in Bergen, Norway (2004) accounted for cases of ME/CFS-like illness following infection with *Giardia enteritis* [38]. Assuming equal exposure risk to common infectious agents for males and females, with higher female dominance, we speculate that genotype and the host response, including hormonal mediation, are important risk factors.

Our finding that being single or separated/divorced is associated with ME/CFS may be reverse causal, as ME/CFS often severely impacts physical health and restricts social functional ability [39]. Other studies have also found that participants with ME/CFS are more likely to be unmarried compared to healthy counterparts [23]. Reverse causality may also be the reason for lower income reported by people with ME/CFS, which have previously discussed [39]. We found an association between reports of family history of anxiety and ME/CFS, but not with personal history of anxiety. It has been reported that people with ME/CFS often have a higher prevalence of psychiatric comorbidities, primarily depression and anxiety disorder [40], and that ME/CFS is associated with higher levels of psychological distress compared with other chronic illness states, such as rheumatoid arthritis [41]; however we did not find a higher reported personal history of either depression or anxiety disorder in people with ME/CFS. We can argue that minor psychiatric morbidity may well reflect the consequences of living with a disabling chronic disease. There is inconsistent evidence whether or not primary psychiatric disorder is a significant risk factor in ME/CFS [40]. One complication with studies of pre-morbid risk in ME/CFS is that ME/CFS patients commonly wait many years to get an affirmative diagnosis, thus studies of pre-diagnostic illness may be detecting psychopathology secondary to the uncertainty of a diagnosis, and “unexplained” symptoms. In addition, given the overlap that exists between the symptoms of ME/CFS and psychiatric disorders, (fatigue, low mood, poor sleep) misdiagnosis may be considerable. In a study of 279 patients referred to a Belgium clinic with suspected chronic fatigue syndrome, 45.2% were diagnosed with a mood or anxiety disorder, yet only 23.3% of the entire cohort eventually received an unequivocal CFS diagnosis [42]. In a UK study of referrals to a specialist CFS treatment centre, out of 260 patient referrals examined, 40% of these did not have CFS but other medical and psychiatric illnesses [43].

### ME/CFS versus MS

ME/CFS cases were shown to be more likely to have a history of colds and other infections 6 months prior to disease onset than MS cases; which is consistent with the findings of comparing ME/CFS with healthy controls, and with the current theories of predisposing/trigger factors (see section above). Tentative links have also been made between MS and human herpes viruses (HHV-6 and EBV) [44]. As in MS, no causal link between one pathogenic agent and ME/CFS has been clearly established.

In addition, there was a larger proportion of people living with partners among people with MS than in people with ME/CFS; and lower income was also reported by people with ME/CFS, which we argue to be explained by reverse causality. This could also be partially related to the fact that people with ME/CFS were younger than MS cases at the time of developing disease symptoms.

### Mild-moderate versus severe ME/CFS

Participants with more severe ME/CFS in our cohort were younger by an average of 4.5 years at disease onset and were more likely to report a family history of neurological problems. The association with age and illness severity may reflect the fact that younger sufferers who go on to have ME/CFS for longer periods, are more likely to have moderate to severe illness presentations. Norris et al. report a large-scale follow-up of adolescents with suspected chronic fatigue syndrome (age 13–18); 75% spontaneously recover within 2–3 years [45], with a quarter with persistent disease. In a previous study, we reported on ME/CFS participants having more pronounced neuro-cognitive symptoms compared with MS participants [46]. The association between ME/CFS and a family history of neurological illness points to genetic risk factors and/or environmental exposure risk; such findings require much more detailed investigation, such as on the confirmation of the diagnosis in the relative and the inclusion of a more formal family history investigation with family pedigrees. We must also consider the ways in which ME/CFS participants recount their symptom experience compared with the ways in which people with MS participants experience illness; ME/CFS patients often have limited medical support, whereas MS is a recognised neurological disease for which there is specialist NHS support, and this may affect the reliability of the information reported by the individual.

### Strengths and limitations

The presence in the final predictive models of variables from all the levels defined in the conceptual approach shows that the occurrence of ME/CFS is the result of a complex multi-factorial process, which includes fixed factors such as age and heredity, and variable factors such as exposure to pathogens. By using a modelling approach involving different factor domains potentially associated with ME/CFS, we have been able to present the relative importance of different risk factors that are often reported in the literature in isolation. Our multivariable analyses helps to capture how different factors jointly contribute to predict ME/CFS, with some factors being distal (e.g. age or income) and some factors being proximal (e.g. recent infection experience). This type of conceptual approach is useful for theorising ME/CFS aetiology, but is biased by the selective inclusion and exclusion of factors investigated. Other risk factors not studied may also be relevant, such as alternative infectious agents, for example. Recall bias is also a major issue, which is likely to be differential, particularly when people with ME/CFS are compared to healthy controls. Data collected from ME/CFS and MS participants relate to a period before they became ill, and there is no equivalent period space for healthy controls, making comparisons challenging between these groups. Nevertheless, healthy control populations offer a reasonable comparison group. Also, the data result from a survey where only a small fraction of the individuals reached by the survey has responded and it is not possible to guarantee or ascertain that this is a representative sample of the targeted population. From the point of view of model building, the conceptual model selected the variables in a manner that aimed to reduce the number of variables in the predictive model; and, by reducing the number of variables we also reduced the impact of missing values on predictive power. However, our model did not consider non-linear terms/ interactions and this may be a reason why the goodness of fit chi squared test reached significance for the comparison with MS, and the specificity was very low (besides the smaller sample size of MS cases in comparison with the healthy controls).

We believe that if the conceptual model we used in this study is applied to well-designed prospective cohorts with larger sample size, some of the limitations described would be overcome, and more significant contributions to knowledge of the factors predictive of ME/CFS could be made.

## Conclusions

Our findings suggest a stronger risk association between exposure to common viral infections (colds/flu) and ME/CFS than seen in the literature. Additionally, we found that a recent history of infection prior to disease onset is associated with ME/CFS. Notable differences in risk profiles were found between participants with ME/CFS and healthy controls and ME/CFS and MS. However, we also found commensurate overlap in some risk factors between all cohorts. This suggests that while ME/CFS may share some similar risks with MS, there are notable differences, particularly the strong association with infection in ME/CFS. Our findings add to the increasing body of evidence on the role infections in the pathogenesis of ME/CFS.

## Supplementary information

**Additional file 1.** Participant Questionnaires.

## Data Availability

The datasets used and/or analysed during the current study are available from the corresponding author on reasonable request.

## Abbreviations

*CCC*: Canadian Consensus Criteria
*CDC*: Centres for Disease Control and Prevention
LSHTM: *London School of Hygiene & Tropical Medicine*
ME/CFS: *Myalgic encephalomyelitis/chronic fatigue syndrome*
MS: *Multiple sclerosis*
NHS: *National Health Service*
NICE: *National Institute for Clinical Excellence*
OR: *Odds ratio*
UKMEB: UK ME Biobank

## References

[1] Ramsay AM, Dowsett EG, Dadswell JV, Lyle WH, Parish JG (1977). Icelandic disease (benign myalgic encephalomyelitis or Royal Free disease). Br Med J.

[2] Holmes GP, Kaplan JE, Gantz NM, Komaroff AL, Schonberger LB, Straus SE (1988). Chronic fatigue syndrome: a working case definition. Ann Intern Med.

[3] Nacul LC, Lacerda EM, Pheby D, Campion P, Molokhia M, Fayyaz S (2011). Prevalence of myalgic encephalomyelitis/chronic fatigue syndrome (ME/CFS) in three regions of England: a repeated cross-sectional study in primary care. BMC Med.

[4] Baker R, Shaw E (2007). Diagnosis and management of chronic fatigue syndrome or myalgic encephalomyelitis (or encephalopathy): summary of NICE guidance. BMJ.

[5] Fukuda K, Straus S, Hickie I, Sharpe M, Dobbins J, Komaroff A (1994). The chronic fatigue syndrome: a comprehensive approach to its definition and study. International chronic fatigue syndrome study group. Ann Intern Med.

[6] Carruthers Bruce M., Jain Anil Kumar, De Meirleir Kenny L., Peterson Daniel L., Klimas Nancy G., Lerner A. Martin, Bested Alison C., Flor-Henry Pierre, Joshi Pradip, Powles A. C. Peter, Sherkey Jeffrey A., van de Sande Marjorie I. (2003). Myalgic Encephalomyelitis/Chronic Fatigue Syndrome. Journal of Chronic Fatigue Syndrome.

[7] Montoya JG, Holmes TH, Anderson JN, Maecker HT, Rosenberg-Hasson Y, Valencia IJ (2017). Cytokine signature associated with disease severity in chronic fatigue syndrome patients. Proc Natl Acad Sci U S A.

[8] Hornig Mady, Montoya José G., Klimas Nancy G., Levine Susan, Felsenstein Donna, Bateman Lucinda, Peterson Daniel L., Gottschalk C. Gunnar, Schultz Andrew F., Che Xiaoyu, Eddy Meredith L., Komaroff Anthony L., Lipkin W. Ian (2015). Distinct plasma immune signatures in ME/CFS are present early in the course of illness. Science Advances.

[9] Albright F, Light K, Light A, Bateman L, Cannon-Albright LA (2011). Evidence for a heritable predisposition to chronic fatigue syndrome. BMC Neurol.

[10] Underhill RA, O'Gorman R (2006). Prevalence of chronic fatigue syndrome and chronic fatigue within families of CFS patients. J Chron Fatigue Synd.

[11] Schlauch KA, Khaiboullina SF, De Meirleir KL, Rawat S, Petereit J, Rizvanov AA (2016). Genome-wide association analysis identifies genetic variations in subjects with myalgic encephalomyelitis/chronic fatigue syndrome. Transl Psychiatry.

[12] Hempel S, Chambers D, Bagnall AM, Forbes C (2008). Risk factors for chronic fatigue syndrome/myalgic encephalomyelitis: a systematic scoping review of multiple predictor studies. Psychol Med.

[13] Reyes M, Nisenbaum R, Hoaglin DC, Unger ER, Emmons C, Randall B (2003). Prevalence and incidence of chronic fatigue syndrome in Wichita, Kansas. Arch Intern Med.

[14] Underhill RA (2015). Myalgic encephalomyelitis, chronic fatigue syndrome: an infectious disease. Med Hypotheses.

[15] Taylor RR, Jason LA (2001). Sexual abuse, physical abuse, chronic fatigue, and chronic fatigue syndrome: a community-based study. J Nerv Ment Dis.

[16] Heim C, Nater UM, Maloney E, Boneva R, Jones JF, Reeves WC (2009). Childhood trauma and risk for chronic fatigue syndrome: association with neuroendocrine dysfunction. Arch Gen Psychiatry.

[17] Lacerda EM, Bowman EW, Cliff JM, Kingdon CC, King EC, Lee J-S (2017). The UK ME/CFS biobank for biomedical research on Myalgic encephalomyelitis/chronic fatigue syndrome (ME/CFS) and multiple sclerosis. Open J Biores.

[18] Victora CG, Huttly SR, Fuchs SC, Olinto MT (1997). The role of conceptual frameworks in epidemiological analysis: a hierarchical approach. Int J Epidemiol.

[19] English indices of deprivation 2015 [https://www.gov.uk/government/statistics/english-indices-of-deprivation-2015].

[20] StataCorp LCC (2018). Stata/IC 15.1 for windows (64-bit x86–64) in*.*, vol. 08 may 2018, 15.1 edn.

[21] Johnston SC, Staines DR, Marshall-Gradisnik SM (2016). Epidemiological characteristics of chronic fatigue syndrome/myalgic encephalomyelitis in Australian patients. Clin Epidemiol.

[22] Bakken I, Tveito K, Gunnes N, Ghaderi S, Stoltenberg C, Trogstad L (2014). Two age peaks in the incidence of chronic fatigue syndrome/myalgic encephalomyelitis: a population-based registry study from Norway 2008 inverted question mark2012. BMC Med.

[23] Faro M, Saez-Francas N, Castro-Marrero J, Aliste L, Fernandez de Sevilla T, Alegre J (2016). Gender differences in chronic fatigue syndrome. Reumatol Clin.

[24] Harbo HF, Gold R, Tintore M (2013). Sex and gender issues in multiple sclerosis. Ther Adv Neurol Disord.

[25] Morris G, Maes M (2013). Myalgic encephalomyelitis/chronic fatigue syndrome and encephalomyelitis disseminata/multiple sclerosis show remarkable levels of similarity in phenomenology and neuroimmune characteristics. BMC Med.

[26] Wang Y, Hunt K, Nazareth I, Freemantle N, Petersen I (2013). Do men consult less than women? An analysis of routinely collected UK general practice data. BMJ Open.

[27] Adamson J, Ben-Shlomo Y, Chaturvedi N, Donovan J (2003). Ethnicity, socio-economic position and gender—do they affect reported health—care seeking behaviour?. Soc Sci Med.

[28] Deale A, Wessely S (2001). Patients' perceptions of medical care in chronic fatigue syndrome. Soc Sci Med.

[29] Katafuchi T, Kondo T, Take S, Yoshimura M (2005). Enhanced expression of brain interferon-alpha and serotonin transporter in immunologically induced fatigue in rats. Eur J Neurosci.

[30] Wuest SC, Mexhitaj I, Chai NR, Romm E, Scheffel J, Xu B (2014). A complex role of herpes viruses in the disease process of multiple sclerosis. PLoS One.

[31] Katz BZ, Collin SM, Murphy G, Moss-Morris R, Wyller VB, Wensaas K-A (2018). The international collaborative on fatigue following infection (COFFI). Fatigue : Biomed Health Behavior.

[32] Chia JK, Chia AY (2008). Chronic fatigue syndrome is associated with chronic enterovirus infection of the stomach. J Clin Pathol.

[33] Clark C, Goodwin L, Stansfeld SA, Hotopf M, White PD (2011). Premorbid risk markers for chronic fatigue syndrome in the 1958 British birth cohort. The British J Psychiat.

[34] Kato K, Sullivan PF, Evengard B, Pedersen NL (2006). Premorbid predictors of chronic fatigue. Arch Gen Psychiatry.

[35] De Bolle L, Naesens L, De Clercq E (2005). Update on human herpesvirus 6 biology, clinical features, and therapy. Clin Microbiol Rev.

[36] Arzomand ML (1998). Chronic fatigue syndrome among school children and their special educational needs. J Chron Fatigue Synd.

[37] Dowset EG, Colby J (1997). Long-term sickness absence due to ME/CFS in UK schools. J Chron Fatigue Synd.

[38] Naess H, Nyland M, Hausken T, Follestad I, Nyland HI (2012). Chronic fatigue syndrome after giardia enteritis: clinical characteristics, disability and long-term sickness absence. BMC Gastroenterol.

[39] Kingdon Caroline C., Bowman Erinna W., Curran Hayley, Nacul Luis, Lacerda Eliana M. (2018). Functional Status and Well-Being in People with Myalgic Encephalomyelitis/Chronic Fatigue Syndrome Compared with People with Multiple Sclerosis and Healthy Controls. PharmacoEconomics - Open.

[40] Afari N, Buchwald D (2003). Chronic fatigue syndrome: a review. Am J Psychiatry.

[41] Katon WJ, Buchwald DS, Simon GE, Russo JE, Mease PJ (1991). Psychiatric illness in patients with chronic fatigue and those with rheumatoid arthritis. J Gen Intern Med.

[42] Mariman A, Delesie L, Tobback E, Hanoulle I, Sermijn E, Vermeir P (2013). Undiagnosed and comorbid disorders in patients with presumed chronic fatigue syndrome. J Psychosom Res.

[43] Newton JL, Mabillard H, Scott A, Hoad A, Spickett G (2010). The Newcastle NHS chronic fatigue syndrome service: not all fatigue is the same. J R Coll Physicians Edinb.

[44] Virtanen JO, Jacobson S (2012). Viruses and multiple sclerosis. CNS Neurol Disord Drug Targets.

[45] Norris T, Collin SM, Tilling K, Nuevo R, Stansfeld SA, Sterne JA (2017). Natural course of chronic fatigue syndrome/myalgic encephalomyelitis in adolescents. Arch Dis Child.

[46] Jain V, Arunkumar A, Kingdon C, Lacerda E, Nacul L (2017). Prevalence of and risk factors for severe cognitive and sleep symptoms in ME/CFS and MS. BMC Neurol.

