# Supplementary material for: A logistic regression analysis of risk factors in ME/CFS pathogenesis
Source: BMC Neurol. 2019 Nov 7;19:275. doi: 10.1186/s12883-019-1468-2 (PMC6839177; doi:10.1186/s12883-019-1468-2)
Supplement: Supplementary file 1 — Additional file 1. Participant Questionnaires. [file 12883_2019_1468_MOESM1_ESM.zip › Participant Quest BASELINE CASES ME&MSR3.docx]

**PARTICIPANT QUESTIONNAIRE**

Project ID:

Today’s date ______/______/________ (DD/MM/YYYY)

1. **Section – Identification**

| - 1. Sex: 1.Male 2.Female |
| --- |
| - 1. Date of birth: ____/______/________ (DD/MM/YYYY) |
| - 1. Post code: _______________ |

1. **Section – Questions about the health of your family**

| - 1. Apart from you, how many children did your biological mother give birth?  1. number of older brothers and sisters ___________ 2. number of younger brothers and sisters_________ 3. number of brothers or sisters the same age as you (e.g. twins, triplets)_________   I do not know | | | | | | |
| --- | --- | --- | --- | --- | --- | --- |
| - 1. Have either of your parents or any of your siblings had any of the following?   If yes, please use the following codes as explained in the instructions: | | | | | | |
| **M** = mother; **F** = father; **B** = brother; **S** = sister | | | | | | |
| 1. High blood pressure? | | 1.Yes |  | _____________ 2.No |  |  |
| 1. Diabetes? | | 1.Yes |  | _____________ 2.No |  |  |
| 1. Heart problem? | | 1.Yes |  | _____________ 2.No |  |  |
| 1. Attacks of wheezing or asthma? | | 1.Yes |  | _____________ 2.No |  |  |
| 1. Allergy, eczema or hay fever? | | 1.Yes |  | _____________ 2.No |  |  |
| Please specify _________________________ | | | | | | |
| 1. Depression? | | 1.Yes |  | _____________ 2.No |  |  |
| 1. Anxiety? | | 1.Yes |  | _____________ 2.No |  |  |
| 1. Cancer? | | 1.Yes |  | _____________ 2.No |  |  |
| Please specify _________________________ | | | | | | |
| 1. Mental health problems? | | 1.Yes |  | _____________ 2.No |  |  |
| Please specify _________________________ | | | | | | |
| - Learning disability? | | 1.Yes |  | _____________ 2.No |  |  |
| Please specify _________________________ | | | | | | |
| - Physical disability? | | 1.Yes |  | _____________ 2.No |  |  |
| Please specify _________________________ | | | | | | |
| 1. ME/CFS | | 1.Yes |  | _____________ 2.No |  |  |
| - Multiple sclerosis (MS) | | 1.Yes |  | _____________ 2.No |  |  |
| - Other neurological problem? | | 1.Yes |  | _____________ 2.No |  |  |
| - Please specify _________________________ | | | | | | |
| - Tuberculosis (TB) | 1.Yes | |  | _____________ 2.No |  |  |
|  |  | |  |  |  |  |
| 1. Any other significant health | 1.Yes | |  | _____________ 2.No |  |  |
| problem(s)? Please specify: _________________________________ | | | | | | |
|  | | | | | | |

1. **Section – Questions about yourself and where you have lived**

| - 1. What is your current marital status? Are you?   1. Single 2. Married 3. Living with partner | | | | | | |
| --- | --- | --- | --- | --- | --- | --- |
| 4. Separated 5. Divorced 6. Widowed | | | | | | |
| - 1. What was your marital status six months before you developed your current symptoms? | | | | | | |
| 1. Single 2. Married 3. Living with partner | | | | | | |
| 4. Separated 5. Divorced 6. Widowed | | | | | | |
| - 1. What are your and your family’s current incomes (after tax) per year from all sources? | | | | | | |
|  | a. Your Income | | | b. Your family’s income n/a | | |
| 1. Nil |  |  |  |  |  |  |
| 2. less than £2,000 |  |  |  |  |  |  |
| 3. £2,000 to £5,999 |  |  |  |  |  |  |
| 4. £6,000 to £9,999 |  |  |  |  |  |  |
| 5. £10,000 to £14,999 |  |  |  |  |  |  |
| 6. £15,000 to £19,999 |  |  |  |  |  |  |
| 7. £20,000 to £29,999 |  |  |  |  |  |  |
| 8. £30,000 to £39,999 |  |  |  |  |  |  |
| 9. £40,000 to £49,999 |  |  |  |  |  |  |
| 10. £50,000 + |  |  |  |  |  |  |
| c. Does that include any benefits you may receive? 1. Yes 2. No  Please specify which benefits you receive: _____________________________ | | | | | | |
| - 1. Six months before developing symptoms, what were your and your family’s incomes per year from all sources? | | | | | | |
|  | a. Your Income | | | b. Your family’s income n/a | | |
| 1. Nil |  |  |  |  |  |  |
| 2. less than £2,000 |  |  |  |  |  |  |
| 3. £2,000 to £5,999 |  |  |  |  |  |  |
| 4. £6,000 to £9,999 |  |  |  |  |  |  |
| 5. £10,000 to £14,999 |  |  |  |  |  |  |
| 6. £15,000 to £19,999 |  |  |  |  |  |  |
| 7. £20,000 to £29,999 |  |  |  |  |  |  |
| 8. £30,000 to £39,999 |  |  |  |  |  |  |
| 9. £40,000 to £49,999 |  |  |  |  |  |  |
| 10. £50,000 + |  |  |  |  |  |  |
| c. Did that include any benefits you may have received? 1. Yes 2. No  Please specify which benefits you received: ______________________________________________________ | | | | | | |

| - 1. What is the highest stage of primary, secondary, or higher education you have reached? | | | | | | | | | | | |
| --- | --- | --- | --- | --- | --- | --- | --- | --- | --- | --- | --- |
| 1. Did not complete primary school | | | | | |  |  | | | | |
| 1. Completed primary school | | | | | |  |  | | | | |
| 1. Secondary school up to GCSE or equivalent | | | | | |  |  | | | | |
| 1. Secondary school up to A-levels or equivalent | | | | | |  |  | | | | |
| 1. Started higher education | | | | | |  |  | | | | |
| 1. Completed higher education | | | | | |  |  | | | | |
| 1. Post-graduate studies | | | | | |  |  | | | | |
| 1. Other: ____________________________ | | | | | |  |  | | | | |
| - 1. Which primary school did you attend? _________________________ n/a   City/Town/Village _____________________ Country__________________ | | | | | | | | | | | |
| - 1. Which secondary school did you attend? ________________________ n/a   City/Town/Village _____________________ Country__________________ | | | | | | | | | | | |
| - 1. In which city/town/village were you born? _____________________   2. In which country were you born? _____________________ | | | | | | | | | | | |
| - 1. What do you consider to be your ethnicity? | | | | | | | | | | | |
| 1. White British | |  | 8. Pakistani | | | | |  |  | | |
| 2. White Irish | |  | 9. Bangladeshi | | | | |  |  | | |
| 3. White other | |  | 10. Chinese | | | | |  |  | | |
| 4. Black African | |  | 11. Other Asian | | | | |  |  | | |
| 5. Black Caribbean | |  | 12. Mixed | | | | |  |  | | |
| 6. Other Black | |  | 13. Other | | | | |  |  | | |
| 7. Indian | |  | Please specify: _____________________ | | | | | | | | |
| - 1. Which type of accommodation do you live in? | | | | | | | | | | | |
| 1. Owned (may be mortgaged) | | | |  | 6. Rent from Housing Association/ | | | | |  |  |
| 2. Rent privately | | | |  | cooperative | | | | |  |  |
| 3. Shared ownership | | | |  | 7. Rent a room or part of a house/flat | | | | |  |  |
| 4. Live rent-free | | | |  | 8. Other | | | | |  |  |
| 5. Rent from local authority (council) | | | |  | Please specify: _____________________ | | | | | | |
| - 1. Please write the city/town/village, postcode (if in the UK) and country you lived:  1. At the time you developed your current symptoms   Town_____________ Post code___________ Country __________   1. Six months before you developed these symptoms:   Town _____________ Post code___________ Country __________ | | | | | | | | | | | |
|  | | | | | | | | | | | |
| - 1. Think about the people living with you:  1. How many adults 18 years old and over live with you (not counting yourself)? __________ 2. How many children under 18 years old live with you? _____________ 3. Does anyone living with you have ME/CFS?   1. Yes 2. Probably, yes 3. No 4. n/a   1. If you answered ‘yes’ or ‘probably yes’, please list the age and sex of those people, and whether they have been diagnosed by a doctor: | | | | | | | | | | | |
| Age | Sex (M or F) | | | | | | | Formally diagnosed? (Y or N) | | | |
| _______ | _______ | | | | | | | _______ | | | |
| _______ | _______ | | | | | | | _______ | | | |
| _______ | _______ | | | | | | | _______ | | | |
| - 1. Have you ever smoked cigarettes regularly? (at least 1 cigarette per day for 1 year or more.)   1. Yes, current smoker 2. Yes, ex-smoker 3. No, never | | | | | | | | | | | |
| - 1. Six months before developing your current symptoms, how much alcohol did you drink on average? (Please refer to instructions to determine number of units per week.)   _______ units per week | | | | | | | | | | | |
| - 1. How much alcohol do you currently drink, on average? (Please refer to instructions to determine number of units per week.)   _______ units per week | | | | | | | | | | | |

1. **Section – Questions about your work**

| - 1. Have you ever been in paid employment or been self-employed?   1. Yes 2. In the past, but not now 3. Never | | | | |
| --- | --- | --- | --- | --- |
| - 1. What is/was your main lifetime occupation? _____ n/a | | | | |
| - 1. What is your present or most recent paid job? If you are or were self-employed, how would you describe what you do?   Job title: __________________________________ n/a | | | | |
| - 1. In this particular job, are/were you? | | | | |
| 1. A manager |  | 4. Self-employed with employees |  |  |
| 2. A foreman or supervisor |  | 5. Self-employed/freelance without employees |  |  |
| 3. An employee (other than |  | 6. Other _______________________ |  |  |
| manager or foreman) 7. Not applicable | | |  |  |
|  | | | | |

| - 1. Please mark the box that best describes your current situation: | | | | |
| --- | --- | --- | --- | --- |
| 1. In full-time paid work |  | 7. In full-time education |  |  |
| 2. In part-time paid work |  | 8. In education less than full-time |  |  |
| 3. Self-employed, full-time |  | 9. Unemployed |  |  |
| 4. Self-employed, part-time |  | 10. Retired |  |  |
| 5. Looking after home or family full-time |  | 11. Sick or disabled |  |  |
| 6. Looking after home or family part-time |  | 12. Unpaid or voluntary work |  |  |
| - 1. How many hours per week do you work and/or study on average, including housework and other unpaid work? ________________ | | | | |
| - 1. Six months before developing symptoms, were you...? (Please mark the box that best describes your situation.) | | | | |
| 1. In full-time paid work |  | 7. In full-time education |  |  |
| 2. In part-time paid work |  | 8. In education less than full-time |  |  |
| 3. Self-employed, full-time |  | 9. Unemployed |  |  |
| 4. Self-employed, part-time |  | 10. Retired |  |  |
| 5. Looking after home or family full-time |  | 11. Sick or disabled |  |  |
| 6. Looking after home or family part-time |  | 12. Unpaid or voluntary work |  |  |
| - 1. Six months before developing symptoms, how many hours per week were you working and/or studying on average, including housework and other unpaid work? ____________ | | | | |
| - 1. Your job title at that time: ____________________________________ n/a | | | | |

1. **Section – Questions about your health history**

| - 1. When did you first notice symptoms*? _____/ _______(MM/YYYY)   ** “Symptoms” refers to any symptoms you associate with the current disease, including fatigue, immune, neurological, or other symptoms.* |
| --- |
| - 1. How did the symptoms start?   1. Suddenly 2. Over time 3. Not sure |
| - 1. Do you think any factor triggered/activated your disease 1.Yes 2.No   If yes, please specify: __________________________________________________ |
| - 1. Compared to when symptoms first started, do you think that you are now… |
| 1. Fully recovered 2.Much better 3. A little better 4.Same  5. A little worse 6. Much worse 7. Don’t know |

| - 1. Six months before you developed symptoms, | | | | | | | |
| --- | --- | --- | --- | --- | --- | --- | --- |
| 1. Was your health?   1. Excellent 2. Good 3. Fair 4. Poor 5. Don’t know | | | | | | | |
| 1. Were you?   1. Very happy 2. Fairly happy 3. Neither happy nor unhappy  4. Not very happy 5. Not at all happy 6. Don’t know | | | | | | | |
| 1. How active were you?   1. Not at all active 2. Rather inactive 3. Neither active nor inactive  4. Rather active 5. Very active 6. Don’t know | | | | | | | |
| - 1. At any time during the **six months before** developing symptoms, did you or were you: | | | | | | | |
| 1. Live on a farm? | 1.Yes |  | 2.No |  | 3. Don’t know |  |  |
| 1. Bitten by a tick? | 1.Yes |  | 2.No |  | 3. Don’t know |  |  |
| 1. Have any vaccinations/immunisations? | 1.Yes |  | 2.No |  | 3. Don’t know |  |  |
| Please specify: _____________________________________________________ | | | | | | | |
| 1. Travel overseas? | 1.Yes |  | 2.No |  | 3. Don’t know |  |  |
| Where? _____________________________________________________ | | | | | | | |
| 1. Exposed to flooding? | 1. Yes |  | 2.No |  | 3. Don’t know |  |  |
| 1. Exposed to radiation? | 1. Yes |  | 2.No |  | 3. Don’t know |  |  |
| Please specify: _____________________________________________________ | | | | | | | |
| 1. Exposed to carbon monoxide? | 1. Yes |  | 2.No |  | 3. Don’t know |  |  |
| 1. Exposed to pesticides? | 1. Yes |  | 2.No |  | 3. Don’t know |  |  |
| 1. Exposed to any other chemicals? | 1. Yes |  | 2.No |  | 3. Don’t know |  |  |
| Please specify (if known): _____________________________________________ | | | | | | |  |
| 1. Have meningitis? | 1.Yes |  | 2.No |  | 3. Don’t know |  |  |
| 1. Have any other serious infection? | 1.Yes |  | 2.No |  | 3. Don’t know |  |  |
| Please specify: _____________________________________________________ | | | | | | | |
| 1. Have an accident where you hit your | 1.Yes |  | 2.No |  | 3. Don’t know |  |  |
| head badly? |  |  |  |  |  |  |  |
| 1. Have a major operation? | 1.Yes |  | 2.No |  | 3. Don’t know |  |  |
| 1. Women only: n/a |  |  |  |  |  |  |  |
| take the (contraceptive) pill? | 1.Yes |  | 2.No |  | 3. Don’t know |  |  |
|  |  |  |  |  |  |  |  |
| 1. Live in a house with gas or oil | 1.Yes |  | 2.No |  | 3. Don’t know |  |  |
| appliances for heating or cooking? |  |  |  |  |  |  |  |
| 1. Live in a house with a gas fire? | 1.Yes |  | 2.No |  | 3. Don’t know |  |  |
|  |  |  |  |  |  |  |  |
| - 1. Have you ever received the BCG | 1.Yes |  | 2.No |  | 3. Don’t know |  |  |
| vaccination for tuberculosis (TB)? |  |  |  |  |  |  |  |
|  |  |  |  |  |  |  |  |

| - 1. Have you had any of the following? Please mark your answer for both “before” and “after” you developed the symptoms and refer to the instructions as needed. | | | | | | | | | | | | | | | |
| --- | --- | --- | --- | --- | --- | --- | --- | --- | --- | --- | --- | --- | --- | --- | --- |
|  | Before developing the symptoms | | | | | | | After developing the symptoms | | | | | | | |
| 1. High blood pressure? | 1.Yes |  | 2.No | |  | |  | 1.Yes | |  | | 2.No |  | |  |
| 1. A heart problem? | 1.Yes |  | 2.No | |  | |  | 1.Yes | |  | | 2.No |  | |  |
| 1. Attacks of wheezing or asthma? | 1.Yes |  | 2.No | |  | |  | 1.Yes | |  | | 2.No |  | |  |
| 1. Allergy, e.g. hay fever or eczema? | 1.Yes |  | 2.No | |  | |  | 1.Yes | |  | | 2.No |  | |  |
| Please specify: _____________________________________________________ | | | | | | | | | | | | | | | |
| 1. Depression? | 1.Yes |  | 2.No | |  | |  | 1.Yes | |  | 2.No | | |  |  |
| 1. Anxiety? | 1.Yes |  | 2.No | |  | |  | 1.Yes | |  | 2.No | | |  |  |
| 1. Particularly high levels of stress? | 1.Yes |  | 2.No | |  | |  | 1.Yes | |  | 2.No | | |  |  |
| 1. Frequent coughs and colds? | 1.Yes |  | 2.No | |  | |  | 1.Yes | |  | 2.No | | |  |  |
| 1. Intolerance to alcohol? | 1.Yes |  | 2.No | |  | |  | 1.Yes | |  | 2.No | | |  |  |
| 1. Intolerance to sugar? | 1.Yes |  | 2.No | |  | |  | 1.Yes | |  | 2.No | | |  |  |
| 1. Any other significant symptoms? | 1.Yes |  | 2.No | |  | |  | 1.Yes | |  | 2.No | | |  |  |
| Please specify: _____________________________________________________ | | | | | | | | | | | | | | | |
|  | | | | | | | | | | | | | | | |
| - 1. Over the last 3 months… | | | |  | |  | | |  | | | | | | |
| a. have you had very dry eyes? | | | | 1.Yes | | 2.No | | | 3. Don’t know | | | | | | |
| b. have you had a very dry mouth? | | | | 1.Yes | | 2.No | | | 3. Don’t know | | | | | | |
|  | | | |  | |  | | |  | | | | | | |
| - 1. Can you now [or could you ever] | | | |  | |  | | |  | | | | | | |
| a. place your hands flat on the floor without bending your knees? | | | | 1.Yes | | 2.No | | | 3. Don’t know | | | | | | |
| b. bend your thumb to touch your forearm? | | | | 1.Yes | | 2.No | | | 3. Don’t know | | | | | | |
| - 1. As a child, did you amuse your friends by contorting your body into strange shapes or could you do the splits? | | | | 1.Yes | | 2.No | | | 3. Don’t know | | | | | | |
| - 1. As a child or teenager, did your kneecap or shoulder dislocate on more than one occasion? | | | | 1.Yes | | 2.No | | | 3. Don’t know | | | | | | |
| - 1. Do you consider yourself ‘‘double-jointed’’? | | | | 1.Yes | | 2.No | | | 3. Don’t know | | | | | | |
|  | | | | | | | | | | | | | | | |

1. **Section – Questions about your current health**

Please fill this out within two days of your blood test appointment (either before or after)

| - 1. Think about how you have been over the last week: |
| --- |
| 1. Is your health?   1. Excellent 2. Good 3. Fair 4. Poor 5. Don’t know |
| 1. Are you?   1. Very happy 2. Fairly happy 3. Neither happy nor unhappy  4. Not very happy 5. Not at all happy 6. Don’t know |
| 1. How active have you been?   1. Not at all active 2. Rather inactive 3. Neither active nor inactive  4. Rather active 5. Very active 6. Don’t know   - 1. During the past month, have you often been bothered by feeling down, depressed, or hopeless? 1. Yes 2. No   2. During the past month, have you often been bothered by little interest or pleasure in doing things? 1. Yes 2. No |
| - 1. Please mark the line with an ‘X’ to describe the severity of pain that you are currently experiencing (think of the last week including today), from no pain at all (mark on the left of the line) to maximum pain (mark on the right of the line).   **Pain as bad**  **No Pain as possible** |
| - - 1. Please mark the line with an ‘X’ to describe the severity of fatigue that you are currently experiencing (think of the last week including today), from no fatigue at all (mark on the left of the line) to maximum fatigue (mark on the right of the line).   **Fatigue as bad**  **No Fatigue as possible** |

| - 1. How much time during the past week... | | | | | | | | | | | | | |
| --- | --- | --- | --- | --- | --- | --- | --- | --- | --- | --- | --- | --- | --- |
|  | **None of the time** | **A little of the time** | | **Some of the time** | | | **A good bit of the time** | | **Most of the time** | | | **All of the time** | |
| Did you feel worn out? 0 1 2 3 4 5  Did you have a lot of energy? 0 1 2 3 4 5  Did you feel tired? 0 1 2 3 4 5  Did you have enough energy to  do the things you wanted to do? 0 1 2 3 4 5  Did you feel full of pep (lively)? 0 1 2 3 4 5 | | | | | | | | | | | | | |
| - 1. During the past week, have you found that… | | | **Disagree 🡨 🡪 Agree** | | | | | | | | | | |
| My motivation is lower when I am fatigued | | | 1 | | 2 | 3 | | 4 | | 5 | 6 | | 7 |
| Exercise brings on my fatigue | | | 1 | | 2 | 3 | | 4 | | 5 | 6 | | 7 |
| I am easily fatigued | | | 1 | | 2 | 3 | | 4 | | 5 | 6 | | 7 |
| Fatigue interferes with my physical functioning | | | 1 | | 2 | 3 | | 4 | | 5 | 6 | | 7 |
| Fatigue causes frequent problems for me | | | 1 | | 2 | 3 | | 4 | | 5 | 6 | | 7 |
| My fatigue prevents sustained physical functioning | | | 1 | | 2 | 3 | | 4 | | 5 | 6 | | 7 |
| Fatigue interferes with carrying out certain duties and responsibilities | | | 1 | | 2 | 3 | | 4 | | 5 | 6 | | 7 |
| Fatigue is among my three most disabling symptoms | | | 1 | | 2 | 3 | | 4 | | 5 | 6 | | 7 |
| Fatigue interferes with my work, family, or social life | | | 1 | | 2 | 3 | | 4 | | 5 | 6 | | 7 |

| - 1. In the past week, how likely were you to doze off or fall asleep in the situations described below, in contrast to just feeling tired? Even if you have not done some of these things recently try to work out how they might have affected you. Use the following scale to choose the most appropriate number for each situation:   0 = would *never* doze  1 = *Slight* chance of dozing  2 = *Moderate* chance of dozing  3 = *High* chance of dozing | | | | | | | | |
| --- | --- | --- | --- | --- | --- | --- | --- | --- |
| **Situation:** | | | **Chance of dozing:** | | | | | |
| Sitting and reading | | |  | |  |  | | |
| Watching tv | | |  | |  |  | | |
| Sitting, inactive in a public place (e.g. a theatre or a meeting) | | |  | |  |  | | |
| As a passenger in a car for an hour without a break | | |  | |  |  | | |
| Lying down to rest in the afternoon when circumstances permit | | |  | |  |  | | |
| Sitting and talking to someone | | |  | |  |  | | |
| Sitting quietly after lunch without alcohol | | |  | |  |  | | |
| In a car, while stopped for a few minutes in traffic | | |  | |  |  | | |
|  | | | | | | | | |
| - 1. Please score how severe your fatigue was when you started having symptoms and over the past week, using the guide in the instructions. If you don’t have or have never had any fatigue, your score will be ‘0’. If your symptoms are very severe, your score will be ‘100’.   Fatigue score when my symptoms started: _____  Fatigue score now: _____ | | | | | | | | |
| - 1. Please tick the boxes using the scale above them to tell us more about which of the symptoms listed below you have had over the past week: | | | | | | | | |
|  | **Absent** | **Mild** | | **Moderate** | | | **Severe** |  |
|  | | | | | | | | |
| *1*. Sore throat |  |  | |  | | |  |  |
| *2*. Symptoms of flu |  |  | |  | | |  |  |
| *3*. Fever or chills |  |  | |  | | |  |  |
| *4*. Tender glands in the neck or arm pit |  |  | |  | | |  |  |
| *5*. Viral infections |  |  | |  | | |  |  |
| *6*. Sensitivities to food, medications, chemicals, |  |  | |  | | |  |  |
| smells/odours, and/or other |  |  | |  | | |  |  |
| *6.a. Intolerance to alcohol (alcohol makes you* |  |  | |  | | |  |  |
| *feel ill/much worse)* |  |  | |  | | |  |  |
| *7*. Feeling ill (malaise) after exertion/activity which |  |  | |  | | |  |  |
| lasts for a long time (more than 24 hours) |  |  | |  | | |  |  |
|  |  |  | |  | | |  |  |
|  | **Absent** | **Mild** | | **Moderate** | | | **Severe** |  |
| *8*. Pain after exertion/activity which lasts for a long |  |  | |  | | |  |  |
| time (more than 24 hours) |  |  | |  | | |  |  |
| *9*. Muscle pain |  |  | |  | | |  |  |
| *10*. Muscle discomfort |  |  | |  | | |  |  |
| *10*.a. Muscle twitching |  |  | |  | | |  |  |
| *11*. Stiffness in the mornings |  |  | |  | | |  |  |
| *11a. Pain in chest or abdomen* |  |  | |  | | |  |  |
| *12*. Pain in two or more joints, without swelling or |  |  | |  | | |  |  |
| redness |  |  | |  | | |  |  |
| *13*. Joint pains moving to different joints without |  |  | |  | | |  |  |
| swelling or redness |  |  | |  | | |  |  |
| *14*. Neck weakness |  |  | |  | | |  |  |
| *15*. Back weakness |  |  | |  | | |  |  |
| *16*. Air hunger, difficulty in breathing or shortness |  |  | |  | | |  |  |
| of breath on exertion/activity |  |  | |  | | |  |  |
| 17. Muscle weakness |  |  | |  | | |  |  |
|  |  |  | |  | | |  |  |
| *18*. Headaches which are new, different or worse |  |  | |  | | |  |  |
| than before the disease started |  |  | |  | | |  |  |
| *19*. Migraine which is different/worse than before |  |  | |  | | |  |  |
| *20*. Unusual sensitivity to light and/or noise |  |  | |  | | |  |  |
| *21*. Temporary disturbance in eyesight |  |  | |  | | |  |  |
| *22*. Tingling or numbness in arms and/or legs |  |  | |  | | |  |  |
| *23*. Loss of balance, unsteadiness on feet when |  |  | |  | | |  |  |
| standing, or inability to focus the vision |  |  | |  | | |  |  |
| *24*. Poor coordination or unsteady movements |  |  | |  | | |  |  |
| (unsteadiness on walking) |  |  | |  | | |  |  |
| *25*. Short term memory problems |  |  | |  | | |  |  |
| *26*. Trouble concentrating |  |  | |  | | |  |  |
| *27*. Confusion or ‘brain fog’ |  |  | |  | | |  |  |
| *28*. Disorientation |  |  | |  | | |  |  |
| *29*. Difficulty understanding things, thinking clearly |  |  | |  | | |  |  |
| *29*.a.Difficulty finding or saying words |  |  | |  | | |  |  |
| *30*. Difficulty retaining/ recalling information |  |  | |  | | |  |  |
| *31*. Slow thinking |  |  | |  | | |  |  |
| *31a.* Difficulty making decisions |  |  | |  | | |  |  |
| *32*. Unrefreshing sleep |  |  | |  | | |  |  |
| *33*. Problems in sleep quality or duration (other than |  |  | |  | | |  |  |
| sleep apnoea), such as insomnia, changing |  |  | |  | | |  |  |
| night for day, awakening during the night |  |  | |  | | |  |  |
| *34*. Marked physical or mental fatigue or exhaustion |  |  | |  | | |  |  |
| after minimal exertion/activity, which lasts for a |  |  | |  | | |  |  |
| long time (more than 24 hours) |  |  | |  | | |  |  |
|  |  |  | |  | | |  |  |

|  | **Absent** | **Mild** | **Moderate** | **Severe** |  |
| --- | --- | --- | --- | --- | --- |
| *35*. Fatigue or exhaustion after levels of activity that |  |  |  |  |  |
| should normally not cause fatigue (i.e. would not |  |  |  |  |  |
| cause fatigue before you got ill) |  |  |  |  |  |
| *36*. Intolerance to exercise (it is difficult to exercise) |  |  |  |  |  |
| *37*. Worsening of symptoms after exertion/activity, |  |  |  |  |  |
| which lasts for a long time (more than 24 hours) |  |  |  |  |  |
| *38*. Worsening of symptoms with stress |  |  |  |  |  |
|  |  |  |  |  |  |
| *39*. Intolerance to (difficulty with) standing on your |  |  |  |  |  |
| feet |  |  |  |  |  |
| *40*. Dizziness while standing up |  |  |  |  |  |
| *41*. Palpitations while standing up |  |  |  |  |  |
| *41a.* Palpitations (feeling that your heart is racing |  |  |  |  |  |
| or pounding) |  |  |  |  |  |
| *42*. Feeling light-headed |  |  |  |  |  |
| *43*. Being extremely pale |  |  |  |  |  |
| *44*. Being unusually sweaty |  |  |  |  |  |
| *44a.* Unusually cold hands or feet |  |  |  |  |  |
| 45. Intolerance to extremes of heat and cold |  |  |  |  |  |
|  |  |  |  |  |  |
| *46*. Feeling sick (nausea) |  |  |  |  |  |
| *46*. a. Irritable bowel symptoms, such as |  |  |  |  |  |
| diarrhoea or constipation, abdominal pain, and |  |  |  |  |  |
| bloating |  |  |  |  |  |
|  |  |  |  |  |  |
| *47*. Bladder problems, such as having a sudden |  |  |  |  |  |
| need to urinate (pass water), urinating more |  |  |  |  |  |
| often than usual, or waking up during the night to |  |  |  |  |  |
| pass water |  |  |  |  |  |
|  |  |  |  |  |  |
| *48*. Decreased sexual interest and/or function |  |  |  |  |  |
|  |  |  |  |  |  |
| *49*. Abnormal appetite or significant changes in |  |  |  |  |  |
| weight (not intentional) |  |  |  |  |  |
| - 1. Women only:   a. On which date did your last period begin? n/a  ______/______/________ (DD/MM/YYYY) | | | | | |
| b. What is the length of your menstrual cycle (e.g., 28 days)? n/a   Nº of days ______ | | | | | |
|  | | | | | |

| Did you complete this questionnaire yourself, or did someone help you? | | | | |
| --- | --- | --- | --- | --- |
| 1. completed myself | | |  |  |
| 1. someone read questions to me | | |  |  |
| 1. someone read and wrote down the answers I gave | | |  |  |
| 1. someone answered the questions for me | | |  |  |
|  | | | | |
| If we have any queries about your questionnaire, would you prefer to: (tick all that apply) | | | | |
| 1. be contacted by phone? | Y |  | | |
| 1. be contacted by letter? |  |  | | |
| 1. be contacted by email? |  |  | | |
| 1. I prefer not to be contacted |  |  | | |
| Best times to be contacted by phone: ___________________________________________ | | | | |

Thank you for completing this questionnaire

Final page of the questionnaire.
